# Idiosyncrasy and Ipecac

**Published:** 1896-11

**Authors:** E. V. Ross

**Affiliations:** Rochester, N. Y.


### IDIOSYNCRASY AND IPECAC.

E. V. Ross, M. D., Rochester, N. Y.

In the September number of The Homœopathic Physician Drs. Selfridge and Martin cite cases of idiosyncrasy in which there was a peculiar susceptibility to the odor of Ipecac. Though quite remarkable, they are put in the shade when compared to a case cited by Gross in his *System of Surgery*, article Idiosyncrasy, Vol. I, p. 44. So remarkable is the case, and coming, as it does, from the pen of a devout allopath of high standing, a believer in all that is material, I may be pardoned if I burden your pages with the following quotation:

"In several persons of my acquaintance, among others a physician, the inhalation or odor of Ipecacuanha invariably excites a violent attack of asthma, generally lasting for two or three days. In the case of the medical practitioner, the perception of this presence of this substance is so keen that if he be in the *third story* of a house on the first floor of which an ordinary dose of the article is compounded, he is instantly seized with spasmodic coughing and wheezing."

It would appear that the susceptibility to the odor of Ipecac is quite marked with some people; it evidently produces quite a perfect picture of asthma. In my hands it has given me and my patients the greatest satisfaction of any remedy I have prescribed. Asthma in itself is not a disease *per se*, but only a symptom and in some cases the main sign of some deep-seated, incurable malady. Hence, Ipec. often acts only in a palliative way. It acts best when administered in a high potency. I prefer the CM.

